# Brain Cortical Complexity Alteration in Amyotrophic Lateral Sclerosis: A Preliminary Fractal Dimensionality Study

**DOI:** 10.1155/2020/1521679

**Published:** 2020-03-19

**Authors:** Jian-Hua Chen, Nao-Xin Huang, Tian-Xiu Zou, Hua-Jun Chen

**Affiliations:** Department of Radiology, Fujian Medical University Union Hospital, Fuzhou 350001, China

## Abstract

**Objective:**

Fractal dimensionality (FD) analysis provides a quantitative description of brain structural complexity. The application of FD analysis has provided evidence of amyotrophic lateral sclerosis- (ALS-) related white matter degeneration. This study is aimed at evaluating, for the first time, FD alterations in a gray matter in ALS and determining its association with clinical parameters. *Materials and Methods*. This study included 22 patients diagnosed with ALS and 20 healthy subjects who underwent high-resolution T1-weighted imaging scanning. Disease severity was assessed using the revised ALS Functional Rating Scale (ALSFRS-R). The duration of symptoms and rate of disease progression were also assessed. The regional FD value was calculated by a computational anatomy toolbox and compared among groups. The relationship between cortical FD values and clinical parameters was evaluated by Spearman correlation analysis.

**Results:**

ALS patients showed decreased FD values in the left precentral gyrus and central sulcus, left circular sulcus of insula (superior segment), left cingulate gyrus and sulcus (middle-posterior part), right precentral gyrus, and right postcentral gyrus. The FD values in the right precentral gyrus were positively correlated to ALSFRS-R scores (*r* = 0.44 and *P* = 0.023), whereas negatively correlated to the rate of disease progression (*r* = 0.44 and *P* = 0.023), whereas negatively correlated to the rate of disease progression (*r* = 0.44 and *P* = 0.023), whereas negatively correlated to the rate of disease progression (

**Conclusions:**

Our results suggest an ALS-related reduction in structural complexity involving the gray matter. FD analysis may shed more light on the pathophysiology of ALS.

## 1. Introduction

Amyotrophic lateral sclerosis (ALS) is a severe, progressive neurodegenerative disease that involves the upper motor neurons of the motor cortex and lower motor neurons of the brain stem and spinal cord [1]. ALS patients present with motor neuron disease [1], such as muscle weakness and spasticity, dysarthria and dysphagia, and hyperreflexia, as well as cognitive alterations, including executive dysfunction, memory decline, and attention deficit [2]. To date, no effective therapy for ALS has been established, and thus, most of the patients die of respiratory dysfunction within 3–5 years [1]. Although ALS has been a major focus in the clinical field, the mechanism underlying this disease is largely unknown.

Neuroscientists have demonstrated cortical dysfunction in ALS. For example, neuropathological studies of ALS have revealed neuronal loss and hyperplasia of astrocyte in the primary motor cortex [3]. Neuroimaging researchers have collectively demonstrated structural, functional, and perfusion abnormalities of the gray matter (GM) in ALS. For instance, based on a high-resolution T1-weighted image, structural MRI analysis revealed cortical atrophy in the precentral cortex, postcentral cortex, insula, cingulate gyrus, and sulcus in ALS [4–7]. Apart from T1-weighted imaging, diffusion-based studies have elucidated a reduction in GM microstructural complexity in the motor and extramotor regions in ALS [8, 9]. Moreover, the structural alteration has been associated with patient cognitive status [5, 6], disease severity [8, 10], and rate of progression [4]. Additionally, resting-state functional MRI studies have suggested that ALS involves a reduction in spontaneous neuronal activity in the sensorimotor cortex and cingulate cortex [11–13], which is also correlated with disease duration and progression rate [12, 13]. In terms of blood flow, PET studies have indicated that ALS patients present decreased regional cerebral blood flow in the sensorimotor cortex, cingulate cortex, and insula [14]. Taken together, cortical abnormalities are a characteristic of ALS that may provide new insights into its underlying mechanism.

Fractal geometry is used to describe the shape complexity of irregular but self-similar objects (e.g., the brain), incorporating detailed information of these shapes [15]. As an index of structural complexity, fractal dimensionality (FD) describes how the structure fills the space [16]. FD analysis has been widely used to quantify cerebral structural complexity alterations and may have more sensitivity to characterize structural alterations than volumetric and gyrification indices [17–19], with small variances and less gender effects [18, 20]. For instance, studies have constantly demonstrated FD reduction of either white matter (WM) or GM in the normal degenerative process [17] and various central nervous system disorders, such as stroke [19], multiple system atrophy [18], multiple sclerosis [21, 22], spinocerebellar ataxia [23], and frontotemporal dementia [20]. In addition, the cerebral FD value is associated with motor function [19] and behavioral manifestation [20]. Using FD analysis, researchers have demonstrated reduced WM structural complexity in ALS, which is also correlated with disease severity [24]. However, there is no study applying FD analysis to investigate the GM structural complexity alterations in ALS. Thus, this study investigated regional FD alterations in ALS and assessed the relationship between regional FD values and clinical characteristics (i.e., disease severity, disease duration, and disease progression rate).

## 2. Materials and Methods

### 2.1. Subjects

Twenty-two patients diagnosed with sporadic ALS and 20 healthy subjects were included in this study. Inclusion criteria for ALS subjects included the diagnosis of definite, probable, or possible ALS and right-handedness. ALS diagnoses were based on the El Escorial criteria [25], whereas disease severity was assessed using the revised ALS Functional Rating Scale (ALSFRS-R). We also recorded the duration of symptoms and the rate of disease progression. The right-handed participants were recruited from the local community as healthy controls, and they were matched with the ALS group, in terms of age and gender. All subjects had the normal mental status (Mini-Mental State Examination (MMSE) score ≥ 27).  Table 1 shows clinical and demographic data gathered in this study. The exclusion criteria were as follows: (1) other neuropsychiatric disorders, including Alzheimer's disease, Parkinson's disease, depression, or epilepsy; (2) receiving psychotropic medications; (3) having other serious disorders, such as respiratory failure, heart failure, and cancer; and (4) contraindication of MRI examination. This evaluation was approved by the Research Ethics Committee of our hospital, China. All of the study participants provided their written informed consent.

### 2.2. MRI Data Acquisition

Three-dimensional T1-weighted images were gathered using a 3T MRI scanner (Prisma, Siemens Medical Systems, Erlangen, Germany), using the magnetization-prepared rapid gradient echo (MPRAGE) sequence as well as the following parameters: TR = 1.61 s, TE = 2.25 ms, flip angle = 8°, matrix = 224 × 224, slice thickness = 1.0 mm (interslice gap = 0 mm), FOV = 224 × 224 mm, and 176 slices.

### 2.3. Calculation of Fractal Dimensionality

Preprocessing of high-resolution T1-weighted images was performed, followed by cortical FD estimation using the standard method in the computational anatomy toolbox (CAT12, http://dbm.neuro.uni-jena.de/cat/) as implemented in Statistical Parametric Mapping software (SPM12, https://www.fil.ion.ucl.ac.uk/spm/). All of the procedures (http://www.neuro.uni-jena.de/cat12/CAT12-Manual.pdf) were conducted using default settings. Image preprocessing comprised correction of bias-field inhomogeneities, followed by segmentation into GM, WM, and cerebrospinal fluid and then normalization using Diffeomorphic Anatomic Registration Trough Exponentiated Lie algebra (DARTEL) algorithm [26]). FD was estimated following the workflow described by Yotter et al. [16] as implemented in CAT12. The workflow allows cortical thickness and central surface reconstructions to be measured in one fully automated step [27]. Then, a spherical harmonic method [28] was employed to reparametrize the cortical surface mesh based on an algorithm that reduces area distortions [29], to repair the topological defects. Finally, the approach of “spherical harmonic reconstructions” proposed by Yotter et al. [16] was used to measure the local fractal dimensionality, which quantifies the cortical surface complexity. Based on spherical harmonic reconstructions [16], the vertex number remains the same for all reconstructed surfaces, which can reduce the effect of individual vertex alignment and avoid the need to progressively regrid the surface. Meanwhile, this approach adopts the information from multiple brain regions when developing the characteristic neuroanatomical signature and allows to calculate a local FD for each vertex within the reconstruction, which is helpful in studies of mental illnesses [16].

Mean FD values were calculated for 152 regions of interest (ROI), which are defined by the Destrieux Atlas [30] (http://surfer.nmr.mgh.harvard.edu/fswiki/CorticalParcellation), with standard procedures for ROI extraction as implemented in the CAT12 toolbox. The estimated FD value in each ROI was compared between the two groups. The statistical threshold was set at a false discovery rate (FDR) corrected value of *P* < 0.05.

### 2.4. Correlation Analysis

The mean FD value of ROIs that survived between-group comparisons was extracted. The correlation between the FD values of these ROIs and clinical parameters (e.g., ALSFRS-R score, duration of symptoms, and rate of disease progression) of the patients was assessed using Spearman correlation analysis. The FDR-corrected *P* value < 0.05 was treated at statistically significant.

## 3. Results

**Figure 1:**
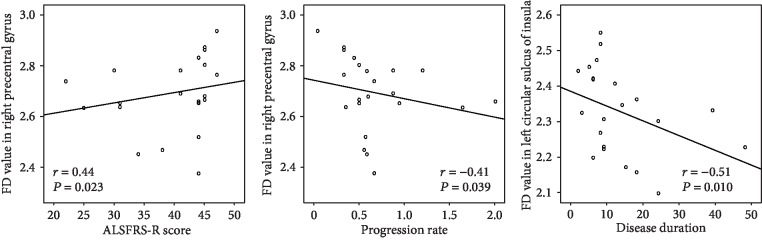
The correlations between FD measurement and ALS patients' clinical parameters.

The ALS patients had a decreased FD value in several cortical regions, including the left precentral gyrus and central sulcus, left circular sulcus of insula (superior segment), left cingulate gyrus and sulcus (middle-posterior part), right precentral gyrus, and right postcentral gyrus.  Figure 1 shows that the FD values in the right precentral gyrus are positively correlated to ALSFRS-R scores (*r* = 0.44 and *P* = 0.023), whereas negatively correlated to the rate of disease progression (*r* = −0.41 and *P* = 0.039). Meanwhile, the FD value in the left circular sulcus of the insula (superior segment) is negatively correlated to disease duration (*r* = −0.51 and *P* = 0.010).

## 4. Discussion

Using FD as an indicator, we discovered reduced cortical complexity in ALS, involving the precentral cortex, postcentral cortex, central sulcus, cingulate gyrus and sulcus (middle-posterior part), and circular sulcus of insula. Correlation analysis suggested that the FD reduction in the motor cortex may be the primary alteration that is correlated with disease severity and progression rate, and extramotor cortical FD reduction might be the secondary change that is related to disease duration.

Cortical complexity in ALS has been examined by a previous study based on the local gyrification index (LGI, the common metric reflecting a cortical folding pattern) [31], which revealed decreased complexity in the occipital cortex but not the key pathological nodes of ALS (e.g., motor cortex) [32]. Methodologically, FD measurement has the inherent advantages and can avoid the drawbacks of LGI estimation (e.g., the dependence on how to define the outer hull, on how to normalize brains to reduce the influence of brain size, and on the noise during surface reconstruction) [16]. These drawbacks could induce the artificial inflation of the surface area without corresponding to the actual anatomy. In addition, several studies have suggested the more sensitivity of FD metric for quantifying cortical complexity alterations than LGI [17–19, 33]. Thereby, FD represents a novel index for quantifying cortical complexity, which may be a favorable complement to other cortical complexity analyses [16], and it is expected that the current study revealed the reduction of structural complexity in more cortical areas (including motor and extramotor regions), as compared to the previous report based on LGI [32]. Thus, applying the FD analysis, we provided a more comprehensive depiction of the structural neural correlates of ALS, in the view of cortical folding complexity.

The disruption of WM connectivity has been well documented in ALS [34, 35], which may directly account for the FD reduction, given that the complexity of cortical folding is thought to be caused by the tension of underlying WM connectivity [32, 36]. In fact, GM FD alterations have been suggested to be secondary to axonal damage in the WM [21]. Axonal swelling and degeneration have been well reported in ALS [37]. Thus, our observation of lower FD in ALS is not unexpected and is concordant with previous research demonstrating decreased LGI in ALS [32]. Nonetheless, a systematic investigation combining histopathological change and FD alteration in the brain in ALS is needed to elucidate the mechanisms underlying FD reduction in ALS.

Our observation of FD reduction in the precentral cortex and central sulcus reflected the morphological abnormality of primary motor cortex in ALS, which agrees with previous investigations that reported the impairments of primary motor cortex in ALS. For example, the ALS-related neuronal loss has been well documented in the primary motor cortex [38]. Furthermore, the accumulating studies have demonstrated regional structure changes (e.g., reduced cortical thickness [4] and gray matter volume [39]) and functional abnormalities (e.g., reduction of spontaneous brain activity at rest [13] and hypoactivation during motor task [7]) in the primary motor-related cortex in ALS. Thus, our finding provided the convergent evidence that the damage to primary motor cortex is the hallmark of ALS, which may underlie the key mechanism about the impaired initiation and inhibition of movement. In addition to motor cortex, several studies have demonstrated the impairments of primary sensory cortex in ALS, such as structural atrophy [40], hypometabolism [41], and functional disturbance [12]. Consistent with these existing reports, we found the involvement of postcentral cortex in ALS, which was reflected by decreased cortical complexity. These abnormalities occurring in the postcentral cortex may convergently explain the sensory dysfunction sometimes seen in ALS patients [1, 42].

We also observed a decline of FD in the cingulate gyrus and sulcus (middle part). In fact, middle cingulum-associated cortical atrophy [13, 43], functional disconnection [44], and hypoperfusion [43] have been observed in ALS. The middle cingulate cortex has widespread connection with the motor cortex and spinal cord, as well as numerous pyramidal neurons [45]. It is responsible for response selection and behavioral reorganization [45]. Therefore, we hypothesize that decreased FD in the middle cingulate cortex may account for motor disability in ALS, which is further supported by a previous study that described a correlation between upper motor neuron function and blood flow of the middle cingulate gyrus in ALS [46].

In addition, we observed a reduction in FD in the posterior cingulate cortex and the insula, suggesting that ALS could result in the damage to structural complexity in these regions and may affect their functions. In line with our results, previous studies have constantly found ALS-associated cortical thinning [4, 6], hypometabolism [41], and reduced resting-state neural activity [13] in the posterior cingulate cortex. Consistent with our finding of FD reduction in the insula, researchers have described insula impairment in ALS, such as cortical thinning [5], energy hypometabolism [47], and decreased cerebral blood flow [43]. Given that the posterior cingulate cortex and insula are functionally associated with several high-order cognitive processes such as memory and attention [48–51], it is speculated that FD reduction in these brain regions may be the neurobiological bases of the cognitive dysfunctions in ALS. It may support the concept that, to some extent, ALS is a motor neuron disease, but which can accompany with the cognitive dysfunction sometimes [6].

This study has several limitations that should be addressed in future investigations. First, the small number of participants may limit the statistical power of our study. Also, the limited sample size prevents us from performing subgroup analysis, which is important for our comprehensive understanding of the clinical heterogeneity of ALS. Therefore, a future study that consists of more participants is needed. Second, we did not perform the specific cognition assessment of ALS patient, which prevents us from directly analyzing the correlation between FD measurement and cognitive performance. Thus, our hypothesis on the relationship between regional FD values and cognitive ability requires further confirmation. Third, previous neuroimaging and neuropathological studies have demonstrated that more widespread regions are implicated in ALS progression [52, 53]. Thus, a longitudinal study investigating the pattern of FD alterations during the course of the disease is warranted.

## 5. Conclusions

The observed FD reduction suggests a decrease in GM structural complexity in ALS, which involves motor-, sensory-, and cognition-related areas. This finding further supports the notion that ALS is a multisystem disease. FD analysis may shed more light on the pathophysiology of ALS.

## Figures and Tables

**Table 1:** Demographic and clinical characteristics of the study participants in this study.

	Healthy controls (*n* = 20)	ALS patients (*n* = 22)	*P* value
Age (years)	54.5 ± 5.5	57.2 ± 10.2	0.27
Sex (males/females)	12/8	15/7	0.58
Site of onset (bulbar/cervical/thoracic/lumbosacral)	—	4/11/1/6	—
Diagnostic category (definite/probable/possible)	—	5/6/11	—
ALSFRS-R score	—	39.7 ± 7.4	—
Disease duration (months)	—	13.5 ± 11.5	—
Disease progression rate	—	0.69 ± 0.45	—

ALS: amyotrophic lateral sclerosis; ALSFRS-R: revised ALS Functional Rating Scale. The rate of disease progression was calculated using the equation: (48-ALSFRS-R)/disease duration. “—” denotes no data available.

## Data Availability

The MRI and clinical data used to support the findings of this study are available from the corresponding author upon request.

## Abbreviations

ALS: Amyotrophic lateral sclerosis
FD: Fractal dimensionality
CAT12: Computational anatomy toolbox
GM: Gray matter
WM: White matter
ALSFRS-R: Revised ALS Functional Rating Scale.
